# Bidirectional, Bimodal Ultrasonic Lamb Wave Sensing in a Composite Plate Using a Polarization-Maintaining Fiber Bragg Grating

**DOI:** 10.3390/s19061375

**Published:** 2019-03-19

**Authors:** Chunfang Rao, Lingze Duan

**Affiliations:** 1 Department of Physics, University of Alabama in Huntsville, Huntsville, AL 35899, USA; Lingze.duan@uah.edu; 2 College of Physics and Communication Electronics, Jiangxi Normal University, Nanchang 330022, Jiangxi, China

**Keywords:** polarization-maintaining Fiber Bragg Grating sensor, Lamb waves, single-mode responses, composite plate

## Abstract

Lamb wave (LW) is well suited for structural health monitoring (SHM) in advanced composites. However, characteristic differences between the symmetric modes and the anti-symmetric modes often add complexity to SHM systems. The anisotropic nature of composite materials, on the other hand, necessitates direction-sensitive sensing. In this paper we report the experimental demonstration of bidirectional (0° and 90°), bimodal (S0 and A0) LW measurement within the frequency range of 20–140 kHz using a polarization-maintaining fiber Bragg grating (PM-FBG) sensor attached to a composite laminated plate. By selectively interrogating the fast and/or the slow axis of the PM-FBG, we show that not only can the sensor respond to LWs propagating along both directions, but the response can also be used to differentiate the two directions. Moreover, the fast axis of the sensor is able to respond to both the S0 and the A0 modes when the sensor is aligned with the wave propagation direction, whereas single S0 mode response can be achieved with the slow axis operating perpendicularly to the wave propagation direction. Such diverse responses indicate the potential of PM-FBGs as versatile multi-parameter SHM detectors, which can effectively address the challenges posed by material anisotropicity and LW mode diversity.

## 1. Introduction

Lamb waves (LWs) have been widely studied in the field of structural health monitoring (SHM) for damage detection in various structures such as pipes, rails, containers, and vessels [1,2,3,4,5]. In recent years, there has been growing interest in LW-based SHM of composite materials due to the increasing use of composites in aerospace, marine and automotive industries [6,7,8,9,10]. LWs can travel over long distances and are highly sensitive to structural damage [3]. However, LWs are intrinsically multi-mode. At any given frequency, there usually coexists at least two fundamental LW modes, namely, the fundamental symmetric (S0) mode and the fundamental anti-symmetric (A0) mode. The two modes exhibit drastically different characteristics and hence are suitable for different types of damage inspections [5,11]. Moreover, selectively generating the S0 or A0 mode proves to be complex and costly in practice [12,13,14,15,16,17,18]. Meanwhile, composite materials post a different challenge to SHM. Since hybrid laminates such as carbon fiber reinforced polymer (CFRP) are usually highly anisotropic, the propagation characteristics of LWs, including velocity, dispersion, mode coupling and attenuation, display a strong directional dependency in such materials [11,19,20]. As a result, a large number of actuators and sensors are usually required to form a distributed array for accurate and comprehensive evaluation of structural health, which has significantly hindered the commercial exploitation of LW-based techniques in SHM [4,21].

Historically, efforts aimed at addressing these two challenges largely followed two separate paths. On the one hand, in order to control system complexity, novel sensing concepts and algorithms were developed to reduce the number of required SHM nodes [3,4,21] or to enhance the multiplexity of sensors [22,23,24]. One example is the development of fiber Bragg grating (FBG) SHM sensors, which allow multiple sensors to be integrated into a single optical fiber and hence simplify system configurations [24,25,26,27,28]. On the other hand, the demonstrations of mode-selective LW sensors in recent years have enabled differentiation of LW modes at the detector end [29,30,31].

Fundamentally, the problems associated with both material anisotropicity and mode diversity can be addressed, or at least mitigated, by a multi-parameter sensor capable of distinguishing not only different LW modes but also waves from different directions. The underlying principle is to increase the degrees of freedom in sensor parameters so that a single sensor can respond distinctively to signals of different natures. The concept of multi-parameter sensing (MPS) has been explored in the context of SHM in the past, primarily with optical fiber sensors. For example, Hu et al. devised an MPS scheme where fiber-optic acoustic generators and FBG sensors were paired together to form two-component sensing nodes [32]. Drissi-Habti et al. demonstrated distributed multi-axial strain sensing by embedding optical fibers in composite specimen with sinusoidal alignments [33]. Meanwhile, FBGs written on polarization-maintaining fibers, also known as PM-FBGs or FBGs on Hi-Bi fibers, have attracted tremendous research interest over the last two decades in the field of optical sensing due to their potential to achieve MPS [34,35,36,37,38,39,40,41]. In particular, several reports have focused on PM-FBG-based multi-axial strain sensing for SHM. For instance, Bosia et al. characterized the response of PM-FBG sensors in a two-dimensional transverse strain field [38]. Abe et al. included both longitudinal and transverse strains as well as temperature in their measurements with PM-FBG [39]. Prabhugoud and Peters later offered a detailed numerical analysis of FBGs written on different types of PM fibers under three-dimensional loading [40]. Mawatari and Nelson developed both linear and nonlinear theoretical models, which were able to predict triaxial strains based on experimentally measured wavelength shift [41]. More recently, Banks and Wang have investigated acousto-ultrasonic sensing using a PM-FBG sensor in a composite plate and reported multi-axis strain measurement [42].

In the work reported here, we extended the concept of MPS into LW sensing and explored the potential of PM-FBGs as bidirectional (longitudinal and transverse), mode-selective LW sensors. The key feature of LW sensing, when compared to all the aforementioned works, is that the strain field oscillates at ultrasonic frequencies rather than remaining static or changing at low frequencies. At such high frequencies, the multi-parameter nature of the measurement takes the forms of mode diversity and wave directionality. In the current study, the response of an PM-FBG to LWs travelling parallel and perpendicular to the axial direction of the sensor was experimentally measured, with both the S0 and the A0 modes excited in a composite laminated plate. It was hoped that such measurements could shed light on the feasibility of PM-FBGs as multi-parameter LW sensors. 

The paper is organized as follows. In Section 2, key characteristics of ultrasonic LWs and PM-FBG strain sensors are briefly reviewed. In Section 3, the experimental setup and methodology are described. The measurement results and the explanation for the mechanism of responses are presented in Section 4. Finally, discussions and conclusions are drawn in Section 5 and Section 6.

## 2. Overview of Lamb Waves and Polarization-Maintaining Fiber Bragg Grating

### 2.1. Ultrasonic Lamb Waves in Composite

**Figure 1:**
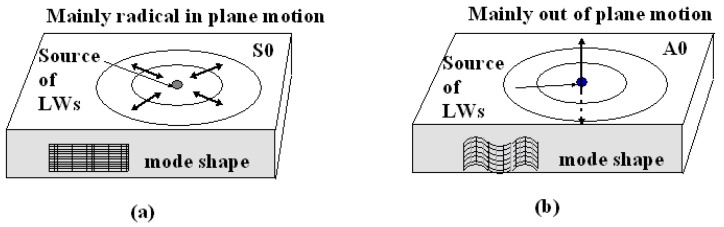
Schematics of mode shape and resulting motions of (**a**) S0 and (**b**) A0 modes at relatively low frequency.

Figure 1 illustrates the main characteristics of particle displacements induced by the A0 and the S0 modes. When the thickness of a plate is less than or comparable to the ultrasonic wavelength, LWs propagate in the sheets of material with propagation vectors parallel to the surface. In general, LWs can produce normal strains along the propagation directions, out-of-plate normal strains, and, in some cases, transversal shear stains in composites [43]. For a symmetric mode, the displacements of particles are symmetric with respect to the neutral axis of the plate. At relatively low frequencies, the S0 mode resembles axial vibrations and its motions are mainly radical within the plane of the plate (Figure 1a). On the other hand, for an anti-symmetric mode, the displacements of particles are anti-symmetrical with respect to the neutral axis. At relatively low frequencies, the A0 mode resembles flexural vibrations and its motions are mainly out of plane (Figure 1b). In this study, the LW frequencies were relatively low (20–140 kHz). Therefore, for the S0 mode, only the normal strain along the wave propagation direction was significant and other normal strains and shear strains could be neglected. On the other hand, for the A0 mode and higher modes, normal strain along the propagation direction, out-of-plate normal strain and transverse shear strain had to be considered [31,44].

### 2.2. PM-FBG-Based Strain Sensors

**Figure 2:**
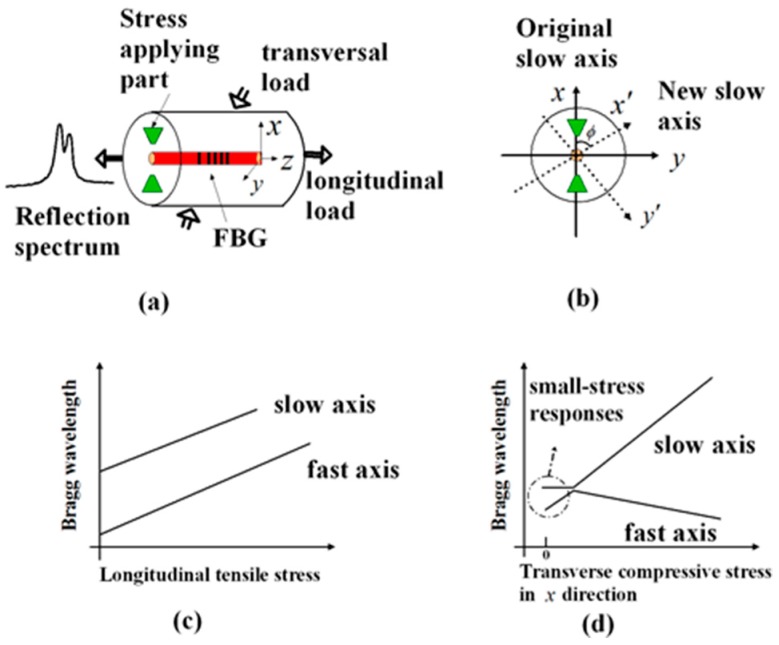
(**a**) Schematic of a Polarization-maintaining Fiber Bragg grating and its spectrum, (**b**) rotation of principal axes under transversal stresses, (**c**) responses of the Bragg wavelength to the longitudinal tensile stress [41] and (**d**) variation of Bragg wavelength originating from the change of effective index of reflection with applied x direction load [40].

Bow-tie fiber is a type of polarization maintaining optical fiber with built-in residual stress in the cladding of the fiber (Figure 2a). Due to the property of birefringence, a propagating mode splits into two orthogonal polarization modes (fast and slow mode). The two principal optical axes of a bow-tie fiber, i.e., the slow and the fast axes, are in the parallel and perpendicular directions relative to the axis established by the stress applying parts (SAP), respectively (Figure 2b). Due to the pre-existing birefringence, the spectral response of the PM-FBG split into two peaks (Figure 2a), which corresponded to the coupling of forward and backward propagations of the two orthogonal polarization modes individually.

When the PM-FBG was loaded along the longitudinal direction, the whole reflection spectrum shifted linearly without distortion (Figure 2c) [41]. However, the PM-FBG response becomes more complicated when other kinds of strains are acting on the sensors. For example, Figure 2d shows the change of Bragg wavelengths originating from the change of effective index of refraction under a compressive stress along the x direction in a PM-FBG fabricated on a bow-tie fiber. Apparently, the two wavelengths moved in opposite directions [40,41]. Meanwhile, if a transverse stress was applied at an arbitrary angle with respect to the unperturbed slow axis *x* (i.e., the fiber was under a shear strain), as shown in Figure 2b, it has been shown that both the slow and the fast axes rotate to become $x^{\prime }$ and $y^{\prime }$, respectively, resulting in changes in the spectra of Bragg reflections. Moreover, the resulted spectral shifts appear to have a nonlinear dependence over the applied transverse stress [38].

## 3. Experimental Setup

**Figure 3:**
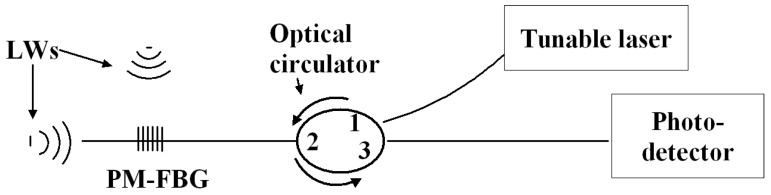
Schematic diagram of edge filtering-based Lamb wave interrogation systems for Polarization-maintaining Fiber Bragg grating.

Our experimental setup is shown in Figure 3. We used an interrogation scheme based on edge filtering [31]. The output from a tunable laser (Photonetics Tunics-plus, Marly-Le-Roi, France) was coupled onto a PM-FBG (Bowtie, 10 mm grating length, and 98% peak reflectivity, by Technica, Atlanta, GA, USA) via an optical circulator. The reflected light was directed to the third port of the circulator and then detected by a photo-detector (New Focus 2053, Newport Corp., Santa Clara, CA, USA). The detector was equipped with a selectable gain and selectable low- and high-pass filters for optimization of the signal level and rejection of the low-frequency drift due to temperature variation in the laboratory. All the optical devices were connected with standard single-mode fibers. When the PM-FBG was subjected to external stress, the shift of the Bragg peaks caused changes in the optical intensity on the photo-detector. The time-dependent output of the detector was recorded by a computer-driven data acquisition system.

**Figure 4:**
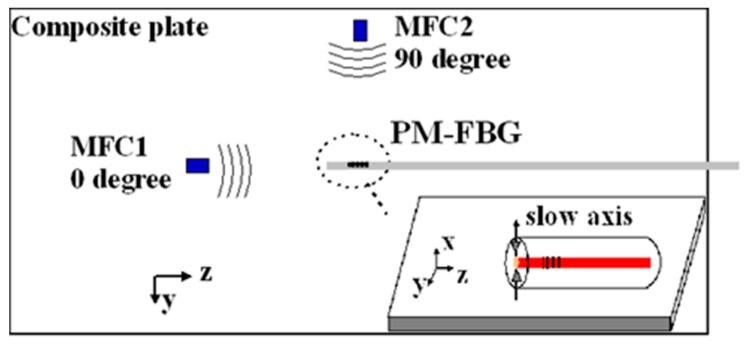
Polarization-maintaining Fiber Bragg grating stuck on the surface of a plate as a Lamb waves (LWs) sensor, two groups of LWs induced by Macro-fiber-composites with propagating directions parallel to and perpendicular to the axis of the sensor individually, and the slow axis of the sensor which was perpendicular to the composite plane.

To study the responses of the PM-FBG under bidirectional and bimodal excitation of LWs, we configured the sensor assembly as shown in Figure 4. Two Macro-fiber-composite (MFC) actuators (M2503-P1, Smart Material Corp., Sarasota, FL, USA) were used as LW generators. They were both 25 mm × 3 mm in dimension and were labelled as MFC1 and MFC2. They were oriented at 0 and 90 degrees with respect to the orientation of the PM-FBG, respectively. Both MFCs and the PM-FBG were epoxied on an oblong composite plate (710 mm × 280 mm), which was made of IM7/8552 material with an 18 cross-ply layup. The MFCs were orientated at 0 and 90 degrees with respect to the zero-degree ply layer. The distance between the center of the MFCs and the PM-FBG was 90 mm. The slow axis of the PM-FBG was set to be perpendicular to the plane of the plate.

During the tests, five-cycled sine bursts were applied periodically to individual MFCs. The bursts were generated via a multifunction I/O interface and were Hanning-windowed to minimize the side lobes. A voltage amplifier were burst to 170 V, and the actuating frequency was adjusted from 20 kHz to 140 kHz for each MFC. Before each test, the laser was carefully set to the wavelength at the half-maximum point of the fast (or slow) axis, which gave the sensor the maximum strain test range. During each experimental cycle, the interrogating laser was first set at the slope of the fast-axis peak to measure the response of the PM-FBG to LWs coming from two different directions. After that, the optical fibers in the experimental setup were rearranged arbitrarily to take into account changes caused by macro bending of fibers. Then the wavelength of the tunable laser was set to the slope of the slow-axis peak and the measurement was repeated.

## 4. Experimental Results

**Figure 5:**
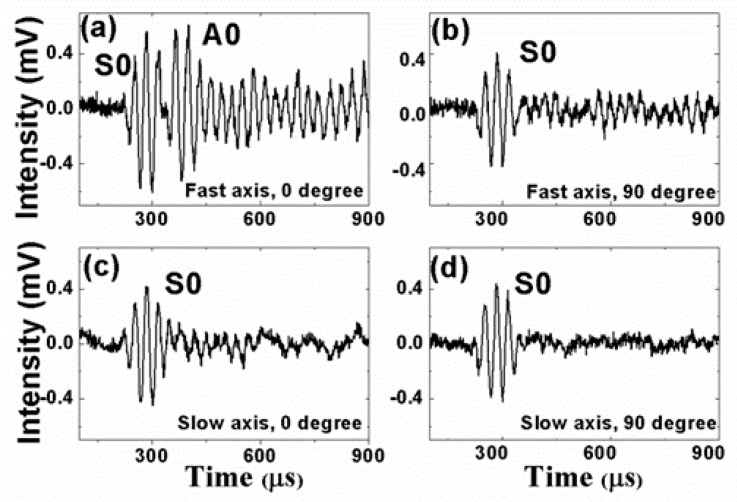
Responses of the Polarization-maintaining Fiber Bragg grating under 30 kHz actuation to (**a**) 0-degree excitation and fast-axis detection; (**b**) 90-degree excitation and fast-axis detection; (**c**) 0-degree excitation and slow-axis detection; and (**d**) 90-degree excitation and slow-axis detection.

**Figure 6:**
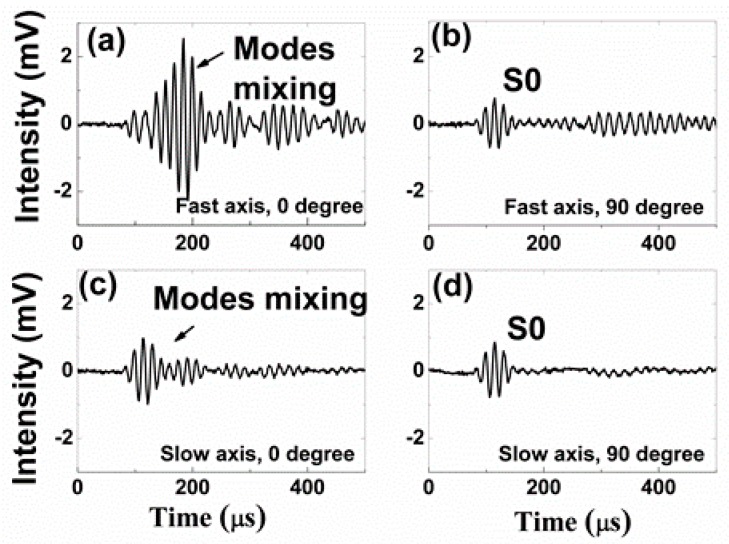
Responses of the Polarization-maintaining Fiber Bragg grating under 60 kHz actuation to (**a**) 0-degree excitation and fast-axis detection; (**b**) 90-degree excitation and fast-axis detection; (**c**) 0-degree excitation and slow-axis detection; and (**d**) 90-degree excitation and slow-axis detection.

**Figure 7:**
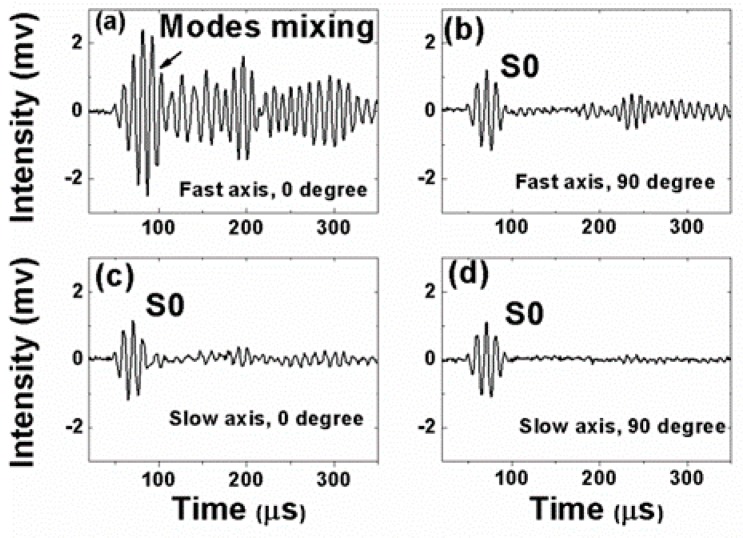
Responses of the Polarization-maintaining Fiber Bragg grating under 90 kHz actuation to (**a**) 0-degree excitation and fast-axis detection; (**b**) 90-degree excitation and fast-axis detection; (**c**) 0-degree excitation and slow-axis detection; and (**d**) 90-degree excitation and slow-axis detection.

**Figure 8:**
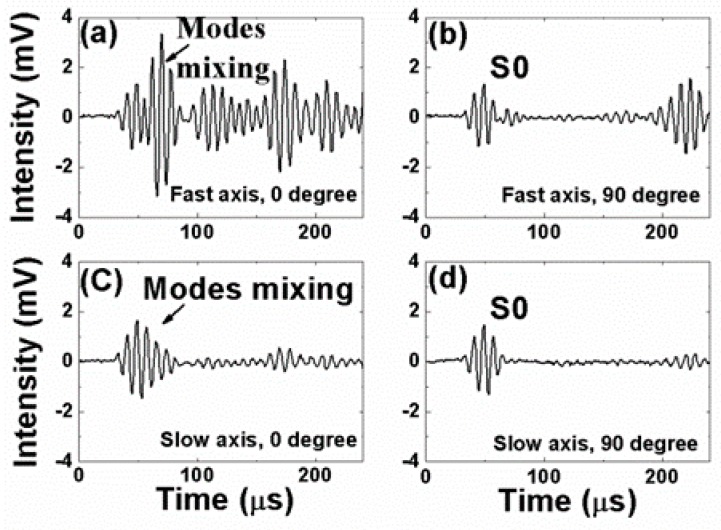
Responses of the Polarization-maintaining Fiber Bragg grating under 120 kHz actuation to (**a**) 0-degree excitation and fast-axis detection; (**b**) 90-degree excitation and fast-axis detection; (**c**) 0-degree excitation and slow-axis detection; and (**d**) 90-degree excitation and slow-axis detection.

Figure 5, Figure 6, Figure 7 and Figure 8 present the PM-FBG responses to the LWs generated by the two MFCs at frequencies of 30 kHz, 60 kHz, 90 kHz, and 120 kHz, respectively. Both the anti-symmetric mode and the symmetric mode were excited by the MFCs because of unimorph actuation. The PM-FBG was able to probe the ultrasonic signals at all of these frequencies. The first two waves (see, for example, Figure 5a) appeared to be S0 and A0. We distinguished the S0 mode from the A0 mode based on the fact that the speed of the S0 mode was greater than that of the A0 mode. The other waves were higher-order mode waves or waves reflected from the edges of the plate.

To gain better insights into the underlying mechanisms, we divided the results into four groups. Group A corresponded to 0-degree excitation (MFC1) and fast-axis detection (Figure 5a, Figure 6a, Figure 7a and Figure 8a). In this case, the PM-FBG was sensitive to both the S0 and the A0 modes and hence both modes were present in the response traces (see, for example, Figure 5a). When the arrival times of the two fundamental modes were too close, such as in the cases of 60 kHz, 90 kHz, and 120 kHz, mode mixing occurred as shown in Figure 6a, Figure 7a and Figure 8a. Moreover, under this condition, the time- dependent responses of the PM-FBG to higher-order modes or the reflected waves were greater in amplitude compared to other conditions. The amplitudes of the A0 modes were larger than that of the S0 modes, which was consistent with the characteristics of LWs [44], and it also proved that the strains remained within the linear scope of the PM-FBG interrogation system in our experiments. Group B contained the results obtained with 90-degree excitation (MFC2) and fast-axis detection (Figure 5b, Figure 6b, Figure 7b and Figure 8b). The S0 modes remained dominant in the responses. However, the sensor response to the A0 modes was significantly reduced. In some traces, such as Figure 6b and Figure 7b, the A0 mode was completely absent. In other traces, such as Figure 8b, only a slight S0-A0 mode mixing could be seen. The responses to higher-order mode waves or reflected waves were still present in this group, albeit at smaller amplitudes than Group A.

Group C included the responses under 0-degree excitation and slow-axis detection (Figure 5c, Figure 6c, Figure 7c and Figure 8c). The PM-FBG was again sensitive to the S0 mode, but A0 mode responses were markedly suppressed. Much weakened S0-A0 mixing could be seen at 60 kHz, and 120 kHz, while the A0 responses became indistinguishable at 30 kHz and 90 kHz. Similar to Group B, higher-order mode waves or reflected waves were detectable but with weaker responses.

Finally, Group D summarized results based on 90-degree excitation and slow-axis detection (Figure 5d, Figure 6d, Figure 7d and Figure 8d). Well-distinguished signals of the S0 modes were detected by the PM-FBG at all frequencies while the A0 modes completely disappeared from all traces. Responses to higher-order mode waves or reflected waves were also highly suppressed, leaving S0 as the only LW mode the PM-FBG sensor responded to.

**Figure 9:**
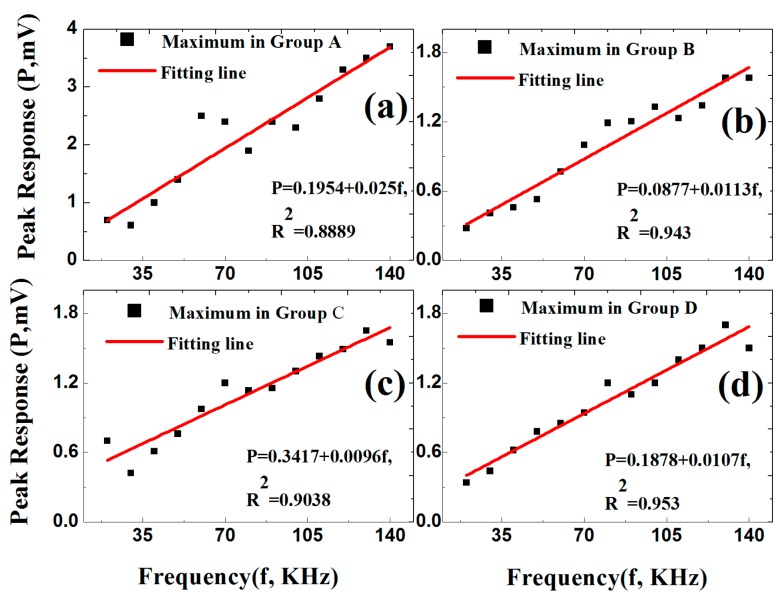
Peak response at each frequency in (**a**) Group A; (**b**) Group B; (**c**) Group C, and (**d**) Group D. The lines are linear fits and the expressions show fitting results.

To evaluate the scales of the sensor response, we took the peak output of the photo-detector as the characteristic value at each frequency in the four groups and found that these values all had linear relations with the actuation frequencies, as shown in Figure 9. The slopes of the linear fittings were 0.025 mV/KHz, 0.0113 mV/KHz, 0.0096 mV/KHz, and 0.0107 mV/KHz, respectively, for the four groups. The worst-case coefficient of determination (R^2^) was 0.8889 for Group A. This was evidently due to the most pronounced mode mixing in Group A. Despite this, the underlying linear relation between sensor response and driving frequency appeared to be robust.

A comprehensive understanding of the aforementioned sensor responses requires extensive simulation of the dynamic strains caused by the ultrasonic waves and the acceleration gradient experienced by the fiber [45], which was beyond the scope of this paper. Here, we only gave a simple and qualitative explanation based on the static responses outlined in Section 2. The dominant strain generated by the S0 mode was the normal strain along the wave propagation direction and the dominant strain generated by the A0 mode was the out-of-plate normal strain perpendicular to the wave propagation direction (see Figure 1). At 0 degrees, A0 mode caused a stress along the *x*-axis. Since this load was caused by the LW, it was reasonable to assume the stress was very small. As shown in Figure 2d, when the stress is small, the fast axis has a significant response to the A0 mode while the slow axis is insensitive to it. On the other hand, since the S0 mode causes in-plane motions along the z direction (longitudinal direction of the fiber), both the fast and the slow axes are able to respond to it (Figure 2c). At 90 degrees, we believed the S0 responses by both the fast and the slow axes were mainly due to the in-plane motion along the z direction rather than the y direction. Since the MFC was close to a point source, even with 90-degree excitation, there could still be some in-plane motion along the longitudinal direction of the fiber, i.e., the z direction. Since an FBG is most sensitive to longitudinal loads, such in-plane motion caused by the S0 mode would nevertheless have generated responses on both the fast and the slow axes of the PM-FBG. On the other hand, for the A0 mode, one would have expected a response similar to the 0-degree case, i.e., some response along the fast axis and no response along the slow axis. However, with 90-degree excitation, both responses were expected to be weaker than 0-degree excitation due to the perpendicular arrangement. Our results appeared to agree with this analysis. The fast axis showed a hint of the A0 mode at some frequencies (e.g., 120 kHz), but in general the response was too low to be picked up by the detector. Meanwhile, the slow axis was completely insensitive to the A0 mode.

## 5. Discussions

The diverse sensor responses shown in Figure 5, Figure 6, Figure 7 and Figure 8 lead to a number of features that can be used in MPS. These features are discussed in the following sections from the perspective of bidirectional detection and bimodal detection.

### 5.1. Bidirectional Lamb Waves Detection

It was clearly evident from the experimental results that a PM-FBG was capable of making bidirectional LW detection. This was especially true when the slow axis of the fiber was used to probe the S0 mode of the LW (Groups C and D). Such a feature could potentially reduce the total number of sensors needed in a SHM network and hence simplify the system configuration.

Moreover, when both symmetric and anti-symmetric modes are excited in a composite plate, a PM-FBG sensor is also able to differentiate between longitudinally propagating LWs and transversely propagating LWs. This can be done by monitoring the fast-axis response, where an absence of the A0 mode indicates that the LWs are likely propagating along the direction perpendicular to the orientation of the fiber (Group A vs. Group B). Such a feature can be very useful in SHM of orthotropic materials such as CFRP, where LWs propagating along two orthogonal major axes are often used to probe possible delamination [27]. The different responses of the fast axis to the A0 mode can potentially serve as a gating signal for automated directional-selection algorithms in a bidirectional sensing scheme.

### 5.2. Bimodal Lamb Waves Detection

It has been shown in previous reports that FBG sensors written on standard telecommunication fibers generally are sensitive to the S0 mode and insensitive to the A0 mode in both metal plates [30] and composite plates [29]. This is a major drawback for FBG-based SHM sensors because the A0 mode is more suitable than the S0 mode for detecting surface cracks due to its flexural nature. Conversely, the S0 mode is extensional in nature and more sensitive to defects inside the structure. Our experimental results showed that a PM-FBG sensor was able to respond to both the A0 and the S0 modes with comparable sensitivity, albeit the sensor had to be aligned with the LW source and the fast-axis response was used (Group A). This bimodal capability potentially allows a single sensor to detect both surface cracks and structural defects provided that proper LW frequencies are selected to avoid mode mixing (e.g., 30 kHz as shown in Figure 5a).

In addition, PM-FBG sensors also display an excellent ability to discriminate the S0 mode from the A0 mode when they are aligned perpendicular to the LW propagation direction and the slow-axis response is used (see Group D). As pointed out in Section 1, such mode-selective sensors can effectively address the problems associated with the multimode nature of LWs. Compared with other mode-selective LW sensing schemes such as FBGs on microstructured fibers [31], the current approach based on PM-FBG is not only simpler to realize but also more effective in terms of mode rejection. Table 1 summarizes the bimodal and bidirectional LW responses by a PM-FBG sensor and compares them with the corresponding responses by a regular FBG sensor.

## 6. Conclusions

In conclusion, the potential of a PM-FBG as a multi-parameter sensor in LW sensing has been explored, for the first time to our knowledge, in a composite laminated plate. The sensor exhibited diverse responses to different modes of the LWs, launched from two different angles (0° and 90°) relative to the sensor orientation within the frequency range of 20 to 140 KHz. By selectively interrogating the fast and/or the slow axis of the PM-FBG, it was shown that bidirectional detection with the ability to differentiate LW propagation directions and bimodal detection with the capability of single-mode sensing (with the S0 mode) could be achieved. Such features can potentially enable multi-functional LW sensors for SHM, leading to simplified system configurations and hence effectively address the problems introduced by the multimode nature of LWs and the anisotropicity of composite materials.

## Figures and Tables

**Table 1:** Bimodal and bidirectional Lamb waves detection of Polarization-maintaining Fiber Bragg grating and corresponding characteristics of fiber Bragg grating.

		0 Degree		90 Degree		Possible Application
**PM-FBG**	**Fast axis**	S0	sensitive	S0	sensitive	Differentiating two propagation directions
		A0	sensitive	A0	weak responses	
	Possible Application		Bimodal LWs detection			
	**Slow axis**	S0	sensitive	S0	sensitive	Bidirectional S0 detection
		A0	week responses	A0	no response	
	Possible Application				Mode-selective sensing	
**FBG [29,30]**	S0		sensitive	S0	insensitive	
	A0		insensitive	A0	insensitive	
